# Association of Plasma Metabolites and Lipoproteins with Rh and ABO Blood Systems in Healthy Subjects

**DOI:** 10.1021/acs.jproteome.2c00375

**Published:** 2022-10-18

**Authors:** Francesca Di Cesare, Leonardo Tenori, Claudio Luchinat, Edoardo Saccenti

**Affiliations:** † Magnetic Resonance Center (CERM), University of Florence, Via Luigi Sacconi 6, Sesto Fiorentino, Firenze 50019, Italy; ‡ Consorzio Interuniversitario Risonanze Magnetiche di Metallo Proteine (CIRMMP), University of Florence, Via Luigi Sacconi 6, Sesto Fiorentino, Firenze 50019, Italy; § Department of Chemistry “Ugo Schiff”, University of Florence, Via della Lastruccia 3, Sesto Fiorentino 50019, Italy; ∥ Laboratory of Systems and Synthetic Biology, Wageningen University & Research, Stippeneng 4, Wageningen 6708 WE, The Netherlands

**Keywords:** metabolomics, lipoproteomics, robust linear models, LDL, HDL

## Abstract

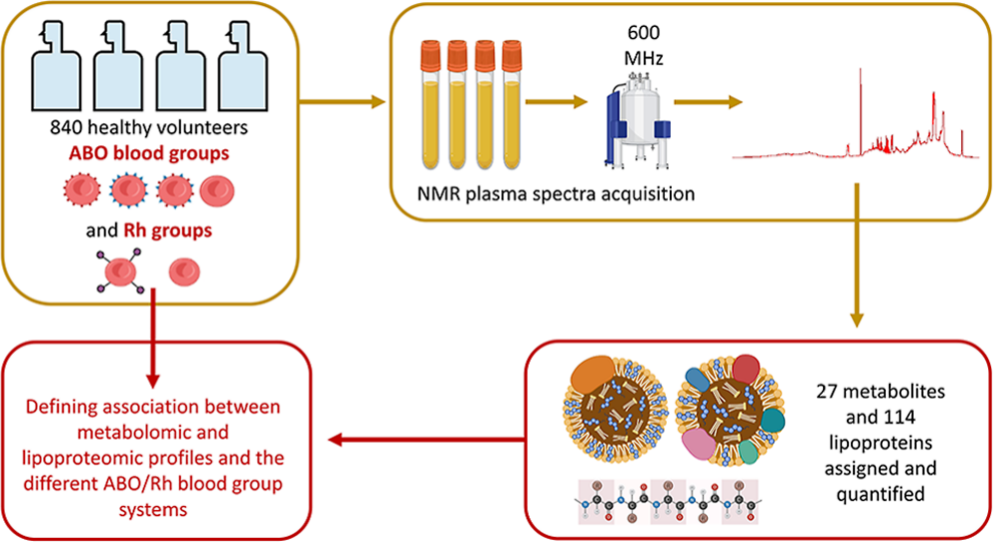

This study investigated the associations between the levels of 27 plasma metabolites, 114 lipoprotein parameters, determined using nuclear magnetic resonance spectroscopy, and the ABO blood groups and the Rhesus (Rh) blood system in a cohort of *n* = 840 Italian healthy blood donors of both sexes. We observed good multivariate discrimination between the metabolomic and lipoproteomic profiles of subjects with positive and negative Rh. In contrast, we did not observe significant discrimination for the ABO blood group pairwise comparisons, suggesting only slight metabolic differences between these group-specific metabolic profiles. We report univariate associations (*P*-value < 0.05) between the subfraction HDL1 related to Apo A1, the subfraction HDL2 related to cholesterol and phospholipids, and the particle number of LDL2 related to free cholesterol, cholesterol, phospholipids, and Apo B and the ABO blood groups; we observed association of the lipid main fraction LDL4 related to free cholesterol, triglycerides, and Apo B; creatine; the particle number of LDL5; the subfraction LDL5 related to Apo B; the particle number of LDL4; and the subfraction LDL4 related to Apo B with Rh blood factors. These results suggest blood group-dependent (re)shaping of lipoprotein metabolism in healthy subjects, which may provide relevant information to explain the differential susceptibility to certain diseases observed in different blood groups.

## 1 Introduction

The most important blood group systems in humans are the ABO and Rh (Rhesus) groups, which consist of carbohydrate moieties, also named human histo-blood group antigens, at the extracellular surface of red blood cell (RBC) membranes.^1^ The human ABO locus is located on chromosome 9 (9q34.2) and has three main allelic forms: (1) allele A that encodes a glycosyltransferase which catalyzes the conversion of the H antigen precursor to the A antigen characterized by the covalent linkage of *N*-acetylgalactosamine to the O antigen; (2) allele B encoding a glycosyltransferase which catalyzes the conversion of the H antigen precursor to the B antigen characterized by the covalent linkage of d-galactose to the O antigen; and (3) allele O that encodes an enzyme with no function, leaving the underlying H antigen precursor structurally unchanged.^2−4^

The Rh system, also known as the D antigen, is based, like the ABO system, on the absence or presence of an antigen on the RBC membrane surfaces; if the antigen D is present, the individual is recognized as Rh positive (Rh^+^), and, if absent, the individual is identified as Rh negative (Rh^–^).^5^

The ABO and Rh systems, which play a fundamental role in transfusion medicine and hematopoietic transplantation, have been associated to the pathogenesis and pathophysiology of various human diseases, such as cardiovascular^6−8^ and oncological diseases,^6,9^ and also have a role in the susceptibility to microorganism, viral, and parasitic infections.^10−13^

While there has been renewed interest on potential associations between ABO/Rh blood groups and the development of specific pathologies,^6,7,14,15^ little is known about the association between the blood metabolomic and lipoproteomic profiles and ABO and Rh blood group systems. The investigation of the existence of blood metabolomic and/or lipoproteomic profiles specific to certain ABO and Rh groups could provide relevant information in the quest for potential blood-specific fingerprints associated with the predispositions to common and chronic pathologies. In this study, we explored the association between the ABO and Rh blood systems and the levels of 27 free circulating plasma metabolites and 114 lipoprotein concentrations and associated parameters, measured using nuclear magnetic resonance (NMR) spectroscopy, in a cohort of 840 healthy blood donor volunteers, using both multivariate and univariate approaches. We report the existence of some weak but significant associations mostly concerning high-density lipoproteins (HDL), low-density lipoproteins (LDL), and apolipoproteins, with blood group systems.

## 2 Materials and Methods

### 2.1 Study Population

The study group consists of 840 adult healthy blood donors, with an overall age range from 19 to 65 years (658 men, with average age of 40.6 ± 10.7 years, and 182 women, with average age of 41.9 ± 12.0 years). Demographic and clinic characteristics are reported in Tables 1 and 2, respectively. Blood donors were enrolled in collaboration with the Tuscany section of the Italian Association of Blood Donors (AVIS) in the Transfusion Service of the Pistoia Hospital (Ospedale del Ceppo, AUSL 3—Pistoia, Tuscany, Italy).

**Table 1:** Demographic and Clinic Characteristics of the Study Population Stratified by ABO Blood Groups

		ABO blood group				
	overall (*n* = 840)	non-O (*n* = 449, 53.5%)	O (*n* = 391, 46.6%)	A (*n* = 330, 39.3%)	AB (*n* = 41, 4.9%)	B (*n* = 78, 9.3%)
**Demographic Parameters**						
age (years)	40.87 ± 11.0	40.9 ± 10.6	40.9 ± 11.4	41.0 ± 10.6	41.1 ± 10.4	40.2 ± 11.1
men (*n*, %)	658, 78.3%	357, 54.3%	301, 45.7%	264, 40.1%	36, 5.5%	57, 8.7%
women (*n*, %)	182, 21.7%	92, 50.6%	90, 49.5%	66, 36.3%	5, 2.8%	21, 11.5%
**Clinical Parameters**						
maximum pressure (mmHg)	123.2 ± 10.7	123.37 ± 10.9	123.1 ± 10.5	123.1 ± 10.6	125.4 ± 13.8	112.9 ± 10.5
minimum pressure (mmHg)	80.6 ± 7.0	80.8 ± 6.9	80.4 ± 7.2	80.5 ± 6.7	82.8 ± 7.5	80.8 ± 7.3
heart rate (bpm)	70.1 ± 5.9	70.2 ± 5.5	70.0 ± 6.3	70.2 ± 5.6	69.3 ± 6.3	71.0 ± 4.7
glycemia (mg/dL)	89.7 ± 11.7	89.8 ± 11.9	89.6 ± 11.5	89.5 ± 12.0	93.3 ± 13.6	89.3 ± 10.5
Chol (mg/dL)	204.1 ± 35.2	205.3 ± 35.2	202.8 ± 35.2	206.0 ± 35.5	208.7 ± 32.9	200.5 ± 35.3
triglycerides (TGs) (mg/dL)	102.3 ± 55.4	104.9 ± 61.6	99.4 ± 47.3	103.8 ± 58.5	111.8 ± 63.3	105.8 ± 73.0
alanine aminotransferase (Unit/L)	23.6 ± 12.1	23.2 ± 13.0	23.1 ± 11.1	23.7 ± 13.3	24.5 ± 12.3	24.5 ± 11.7
hematocrit (HCT) (dL/dL(%))	43.5 ± 3.2	43.3 ± 2.6	43.6 ± 3.7	43.2 ± 2.6	44.0 ± 2.3	43.6 ± 2.9
white blood cells (WBCs)(10^3^/μL)	6.2 ± 4.1	6.3 ± 5.2	6.1 ± 2.1	6.1 ± 1.5	5.6 ± 1.2	7.5 ± 12.2
red blood cells (RBCs) (10^6^/μL)	5.0 ± 0.5	5.0 ± 0.4	5.1 ± 0.6	5.0 ± 0.4	5.1 ± 0.3	5.1 ± 0.4
hemoglobin (g/dL)	15.0 ± 1.4	14.9 ± 1.1	15.0 ± 1.8	14.9 ± 1.1	15.3 ± 1.0	15.1 ± 1.2
mean corpuscular volume (MCV) (fL)	86.8 ± 4.0	86.9 ± 3.9	86.6 ± 4.0	87.1 ± 3.8	86.5 ± 3.3	86.2 ± 4.4
platelets (10^3^/μL)	227.7 ± 48.3	227.8 ± 49.3	227.5 ± 47.2	228.3 ± 49.9	221.2 ± 42.5	229.2 ± 50.2

**Table 2:** Demographic and Clinic Characteristics of the Study Population Stratified by the Rh Blood Group System

		Rh blood group system	
	overall (*n* = 840)	Rh^+^ (*n* = 710, 84.5%)	Rh^–^ (*n* = 130, 15.5%)
**Demographic Parameters**			
age (years)	40.9 ± 11.0	40.7 ± 10.3	41.7 ± 11.1
men (*n*, %)	658, 78.3%	560, 85.1%	98, 14.9%
women (*n*, %)	182, 21.7%	150, 82.4%	32, 17.6%
**Clinic Parameters**			
maximum pressure (mmHg)	123.2 ± 10.7	123.0 ± 10.9	124.4 ± 10.7
minimum pressure (mmHg)	80.6 ± 7.0	80.5 ± 7.1	81.0 ± 7.0
heart rate (bpm)	70.1 ± 5.9	70.4 ± 6.6	69.0 ± 5.7
glycemia (mg/dL)	89.7 ± 11.7	90.1 ± 10.9	87.9 ± 11.8
Chol (mg/dL)	204.1 ± 35.2	204.3 ± 32.3	203.0 ± 35.8
triglycerides (TGs) (mg/dL)	102.3 ± 55.4	102.3 ± 47.6	102.5 ± 56.8
alanine aminotransferase (Unit/L)	23.6 ± 12.1	23.8 ± 12.1	22.2 ± 12.1
hematocrit (HCT) (dL/dL(%))	43.5 ± 3.2	43.5 ± 2.8	43.2 ± 3.2
white blood cells (WBCs)(10^3^/μL)	6.2 ± 4.1	6.3 ± 1.4	5.8 ± 4.4
red blood cells (RBCs) (10^6^/μL)	5.0 ± 0.5	5.0 ± 0.4	5.0 ± 0.5
hemoglobin (g/dL)	15.0 ± 1.4	15.0 ± 1.2	14.8 ± 1.5
mean corpuscular volume (MCV) (fL)	86.8 ± 4.0	86.9 ± 4.0	86.0 ± 3.9
platelets (10^3^/μL)	227.7 ± 48.3	229.4 ± 44.1	218.1 ± 48.8

According to the Italian guidelines for blood donation (Annex III of the Decree of the Italian Ministry of Health dated 2 November 2015),^16^ blood donors must not have (had) infectious, chronic, and/or common diseases before donation, surgery within 3 months before donation, endoscopic exams within 4 months before donation, current menstruation, pregnancy within 12 months before donation, and abortion within 4 months before donation; they had not participated in sport activity within 24 h before donation; and they had not taken drugs within 1 week before donation. For further details, see previous publications.^17−19^

### 2.2 Ethics Statement

The study adheres to the directives of the Declaration of Helsinki (1964).

### 2.3 Sample Preparation and ABO and Rh Determination

Plasma samples were obtained after overnight fasting and, after collection, were stored at −80 °C and handled according to standard operating procedures.^20^ The ABO and Rh blood groups were determined by standard procedures using agglutination techniques.^21^

### 2.4 NMR Analysis

The mono-dimensional nuclear Overhauser effect spectroscopy (NOESY) ^1^H spectra of plasma samples were acquired using a Bruker 600 MHz spectrometer (Bruker BioSpin s.r.l., Germany), operating at 600.13 MHz. For more details about NMR sample preparation, acquisition, and spectral processing, we refer the reader to the original publications.^17,22^

Twenty-seven (27) metabolites were assigned and identified using in-house software developed based on standard line-shape analysis methods and with the help of matching routines of AMIX 7.3.2 (Bruker BioSpin) in combination with the BBIOREFCODE (Version 2–0–0; Bruker BioSpin) reference database and published literature (when available). In the Supporting Information (Figure S1), the assignment of a plasma spectrum is shown. The relative concentration of each metabolite was calculated by integrating the signals in the spectra; 114 lipoprotein fractions and subfractions were assigned and quantified using the AVANCE IVDr [Clinical Screening and In Vitro Diagnostics (IVD) research with B.I. Methods, Bruker BioSpin].^23^ The direct integration of NMR signals was carried out. A list of metabolites and lipoprotein fractions and subfractions and lipids is given in Table 3.

**Table 3:** List of Metabolites and Lipoprotein Fractions and Subfractions Analyzed^a^

metabolites	lipid fractions and subfractions				
3-hydroxybutyrate	MP TG	LMF Free Chol–IDL	Subfr PL–VLDL 2	Subfr Apo B–LDL 1	Subfr Apo A2–HDL 3
acetate	MP Chol	LMF Free Chol–LDL	Subfr PL–VLDL 3	Subfr Apo B–LDL 2	Subfr Apo A2–HDL 4
alanine	MP LDL–Chol	LMF Free Chol–HDL	Subfr PL–VLDL 4	Subfr Apo B–LDL 3	
arginine + lysine	MP HDL–Chol	LMF PL–VLDL	Subfr PL–VLDL 5	Subfr Apo B–LDL 4	
adenosine nucleotide + inosine monophosphate	MP Apo A1	LMF PL–IDL	Subfr TG–LDL 1	Subfr Apo B–LDL 5	
citrate	MP Apo A2	LMF PL–LDL	Subfr TG–LDL 2	Subfr Apo B–LDL 6	
creatine	MP Apo B100	LMF PL–HDL	Subfr TG–LDL 3	Subfr TG–HDL 1	
creatinine	CFLDL–Chol/HDL–Chol	LMF Apo A1–HDL	Subfr TG–LDL 4	Subfr TG–HDL 2	
formate	CFApo A1/Apo B100	LMF Apo A2–HDL	Subfr TG–LDL 5	Subfr TG–HDL 3	
fumarate	PN Total PN	LMF Apo B–VLDL	Subfr TG–LDL 6	Subfr TG–HDL 4	
glucose	PN VLDL	LMF Apo B–IDL	Subfr Chol–LDL 1	Subfr Chol–HDL 1	
glutamate	PN IDL	LMF Apo B–LDL	Subfr Chol–LDL 2	Subfr Chol–HDL 2	
glutamine	PN LDL	Subfr TG–VLDL 1	Subfr Chol–LDL 3	Subfr Chol–HDL 3	
glycine	PN LDL 1	Subfr TG–VLDL 2	Subfr Chol–LDL 4	Subfr Chol–HDL 4	
histidine	PN LDL 2	Subfr TG–VLDL 3	Subfr Chol–LDL 5	Subfr Free Chol–HDL 1	
isoleucine	PN LDL 3	Subfr TG–VLDL 4	Subfr Chol–LDL 6	Subfr Free Chol–HDL 2	
lactate	PN LDL 4	Subfr TG–VLDL 5	Subfr Free Chol–LDL 1	Subfr Free Chol–HDL 3	
leucine	PN LDL 5	Subfr Chol–VLDL 1	Subfr Free Chol–LDL 2	Subfr Free Chol–HDL 4	
mannose	PN LDL 6	Subfr Chol–VLDL 2	Subfr Free Chol–LDL 3	Subfr PL–HDL 1	
methionine	LMF TG–VLDL	Subfr Chol–VLDL 3	Subfr Free Chol–LDL 4	Subfr PL–HDL 2	
phenylalanine	LMF TG–IDL	Subfr Chol–VLDL 4	Subfr Free Chol–LDL 5	Subfr PL–HDL 3	
proline	LMF TG–LDL	Subfr Chol–VLDL 5	Subfr Free Chol–LDL 6	Subfr PL–HDL 4	
pyruvate	LMF TG–HDL	Subfr Free Chol–VLDL 1	Subfr PL–LDL 1	Subfr Apo A1–HDL 1	
tyrosine	LMF Chol–VLDL	Subfr Free Chol–VLDL 2	Subfr PL–LDL 2	Subfr Apo A1–HDL 2	
unknown 1	LMF Chol–IDL	Subfr Free Chol–VLDL 3	Subfr PL–LDL 3	Subfr Apo A1–HDL 3	
unknown 2	LMF Chol–LDL	Subfr Free Chol–VLDL 4	Subfr PL–LDL 4	Subfr Apo A1–HDL 4	
valine	LMF Chol–HDL	Subfr Free Chol–VLDL 5	Subfr PL–LDL 5	Subfr Apo A2–HDL 1	
	LMF Free Chol–VLDL	Subfr PL–VLDL 1	Subfr PL–LDL 6	Subfr Apo A2–HDL 2	

a Abbreviations used: Subfr, subfraction; Chol, cholesterol; MP, main parameter; CF, calculated figure; TG, triglycerides; LMF, lipoprotein main fraction; PL, phospholipids; and PN, particles number.

#### 2.4.1 Data Pre-processing

Only clinical variables with less than 20% missing data were considered; missing data were imputed using a Random Forest (RF) approach as implemented in R package missForest,^24^ using the default parameters. Variables that have been imputed and the percentage of imputation for that specific variable are glycemia (19.6%), maximum pressure (3.7%), minimum pressure (3.8%), heart rate (4.3%), cholesterol (Chol, 17%), triglycerides (TG, 17.8%), alanine aminotransferase (ALT, 0.1%), and hematocrit (HCT, 0.1%).

All metabolite concentrations and lipoproteomic parameters were square-root-transformed before analysis to adjust for heteroscedasticity.^25^

### 2.5 Statistical Analysis

#### 2.5.1 Two-Proportion Z-Test

The observed percentages of ABO and Rh blood groups in the study population were compared with those observed in the general Italian population^26,27^ using a two-proportion *Z*-test.^28^ Results are reported with 95% confidence intervals (CIs).

#### 2.5.2 Exploratory Data Analysis

Principal component analysis (PCA)^29,30^ was used to explore data patterns. Data was scaled to unit variance before analysis.

#### 2.5.3 Predictive Modeling

The Random Forest (RF) algorithm^31−33^ was employed for pairwise classification of metabolite and lipoproteomic profiles of subjects with different ABO and Rh blood groups. The following comparisons were performed: A *versus* AB, AB *versus* B, A *versus* B, A *versus* O, AB *versus* O, B *versus* O blood groups, and Rh^–^*versus* Rh^+^ groups.

To reduce the potential bias due to the unbalanced number of subjects per group, we implemented a resampling scheme with *K* = 100 resampling, considering the sex distribution. In this procedure, we selected, for each pairwise comparison (*i.e.*, Rh^+^*vs* Rh^–^, A *vs* AB, etc), an equal number of individuals, stratified by sex; in more detail, 85% of the subjects per balanced group were randomly selected at each iteration step; basically, for each comparison, 100 different RF models were built.

The model quality statistics, including the accuracy, sensitivity, specificity, and area under the curve (AUC), are given as average values over the *K* = 100 models with the corresponding 95%. Quality statistics were calculated according to standard definitions.^34^

#### 2.5.4 Permutation Test

The statistical significance of the RF classification models was determined with a permutation test using *M* = 1000 permutation. *P*-values were calculated by comparing the value *model*_*0*_, obtained from the original, and non-permuted data with the values *model*_*1*_, *model*_*2*_, ..., *model*_*M*_ obtained from the *M*-times permutation-test. The *P*-value for a specific quality measure is calculated as follows^35^
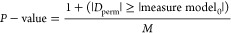
1where |*D*_perm_| is the number of permuted models for which a given quality measure is larger or equal to the quality measure from the original (non-permuted) model (*model*_*0*_).

#### 2.5.5 Robust Linear Regression

The association between plasma metabolites, lipids, and lipoproteins and the ABO and Rh groups was determined by linear regression models,^36,37^ according to the following formula
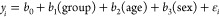
2where *y*_*i*_ is the abundance/concentration of the *i*th molecular feature (metabolite or lipid/lipoprotein), *b*_*i*_ is the estimated regression coefficient that quantifies the association between the ABO/Rh blood groups, adjusted for age and sex, and the molecular features, and ε_*i*_ is the residual part not accounted for by the other terms. To reduce the influence of outliers and high leverage points on the regression solutions, we used a robust version for the linear regression, where the fitting is done by iterated re-weighted least squares.^36,37^ For each model, post-hoc pairwise comparisons among ABO blood group systems were also performed using the estimated marginal means.^38^

The Benjamini–Hochberg method^39^ was used to correct for multiple testing.

### 2.6 Software

All calculations were performed in R (version 4.0.3).^40^ The function “missForest”, implemented in the missForest package, was used to impute the missing data.^24^ RF models were built using the “randomForest” function, implemented in the R package RF,^31,41^ growing a decision forest composed of 1000 trees, using default parameters. To estimate the significance of importance metrics for the RF models by the 1000-times permutation of the response variable, the “importance” function, implemented in the R package rfPermute, was used.^42^ The function “rlm”, implemented in the MASS R package, was used to perform the robust fitting of linear models.^43^ Default parameters were used. The function “emmeans”, implemented in the emmeans R package,^38,44^ was used to compute comparisons among specified factors and/or factor combinations in the linear models.

### 2.7 Data Availability

NMR spectra and associated clinical data are available in the MetaboLights repository^45^ (http://www.ebi.ac.uk/metabolights) with accession number MTBLS147. Data on lipoprotein fractions and subfractions are available at the NIH Common Fund’s National Metabolomics Data Repository (NMDR) the Metabolomics Workbench (https://www.metabolomicsworkbench.org), where it has been assigned Project ID ST001785. The data can be accessed directly *via* its Project DOI: http://dx.doi.org/10.21228/M8470J.

## 3 Results and Discussion

### 3.1 Distribution of ABO and Rh Blood Group Systems in Tuscany

Clinical and demographic characteristics of the study subjects, divided by ABO and Rh groups, are given in Tables 1 and 2, respectively. The list of the metabolites and lipoproteins assigned and quantified is reported in Table 3.

The distribution of the ABO blood groups among subjects (all original from the Pistoia area in Tuscany, Italy), as shown in Table 4, is in line with the distribution of the general population living in Italy.^27^ In particular, the distribution of A, B, and O groups in the study cohort is similar to the distribution observed in Italy, except for the AB group (*P*-value = 0.006).

**Table 4:** Results of the Two-Proportion *Z*-Test of ABO Blood Groups and Rh Blood Group System^a^

ABO blood groups	number of individuals (*n*)	ABO distribution (%)	95% CI	ABO distribution in Italy (%)	adjusted *P*-value
A	330	39.3	36.0–43.0	41.0	0.62
AB	41	4.9	4.0–7.0	3.0	0.006
B	78	9.3	7.0–11.0	11.0	0.34
O	391	46.6	43.0–50.0	46.0	0.75

Rh blood system	number of individuals (*n*)	Rh distribution (%)	95% CI	Rh distribution in Italy (%)	adjusted *P*-Value
Rh^+^	710	84.5	82.0–87.0	85.0	0.74
Rh–	130	15.5	13.0–18.0	15.0	0.74

a In the table, the number of individuals per blood group, the ABO distribution (%) and the Rh blood group system distribution (%) of our population, the 95% CI, the ABO distribution (%), and the Rh blood group system distribution (%) in Italy, and the adjusted *P*-value of the two-proportion *Z*-test are reported.

The proportion of Rh^+^ and Rh^–^ subjects is not different from the general Italian population (see Table 4).^26^

### 3.2 Exploration and Discrimination of Metabolites, Lipids, and Lipoproteins Associated with ABO and Rh Blood Groups

**Figure 1:**
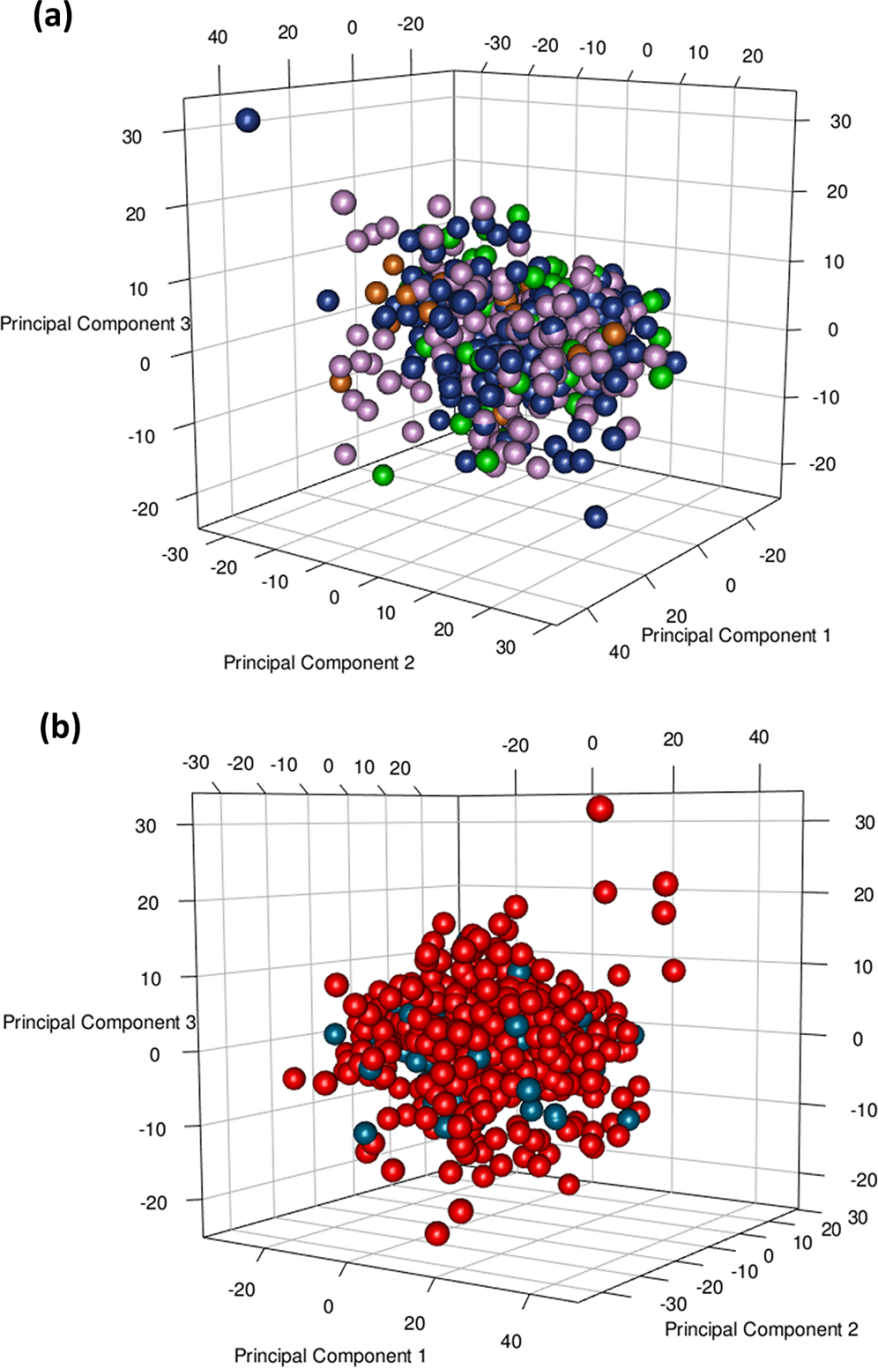
(a) PCA model score plot [PC1 (11.4%) *versus* PC2 (9.2%) *versus* PC3 (5.9%)]. Each dot represents a single metabolic profile with colors denoting different groups of patients: *n* = 330, A blood group subjects (violet dots); *n* = 41, AB blood group subjects (dark orange dots); *n* = 78, B blood group subjects (green dots); and *n* = 391, O blood group subjects (dark blue dots). (b) PCA model score plot [PC1 (11.4%) *versus* PC2 (9.2%) *versus* PC3 (5.9%)]. Each dot represents a single metabolic profile colored by the different groups of patients: *n* = 710, Rh^+^ blood group subjects (red dots); and *n* = 130, Rh^–^ blood group subjects (light blue dots).

To explore comprehensively the metabolomic and lipoproteomic profiles (consisting of 27 metabolites and 114 lipoproteins) associated with the ABO and Rh blood group systems, we applied PCA on the *n* = 840 plasma samples. The PCA score plot in Figure 1a shows no clear separation among the A, AB, B, and O groups, suggesting that metabolic differences are too subtle to be resolved using an unsupervised multivariate approach. A similar lack of separation can be observed in the case of Rh^+^ and Rh^–^ profiles (Figure 1b).

RF classification was applied to investigate whether subjects with different ABO and Rh blood group systems could be discriminated from the metabolic and lipoproteomic profiles. The results of the RF classification for ABO and Rh blood groups are given in Tables 5 and 6, respectively. Overall, we obtained extremely weak classification models to discriminate between the different ABO groups: A *versus* AB groups (AUC = 0.562, *P*-value = 0.04), A *versus* B groups (AUC = 0.514, *P*-value = 0.03), AB *versus* B groups (AUC = 0.535, *P*-value = 0.03), A *versus* O groups (AUC = 0.557, *P*-value = 0.01), and AB *versus* O groups (AUC = 0.568, *P*-value = 0.03). A non-significant predictive model (AUC = 0.503, *P*-value = 0.09) was obtained for the discrimination of B *versus* O groups (see Table 5). These results suggest that the metabolic and lipoproteomic profiles, as a whole, may be weakly associated with the ABO groups; why this is the case is not clear: a lack of association may be a consequence of the limited sample size, or relevant associations may be limited to a few metabolites and/or lipoprotein/lipid features.

**Table 5:** Mean Values of Accuracy, Specificity, Sensitivity, and AUC of RF Models Built Comparing the ABO Blood Groups, A *versus* AB Subjects, A *versus* B Subjects, A *versus* O Subjects, AB *versus* O Subjects, and B *versus* O Subjects

	mean accuracy % (95% CI and *P*-value)	mean specificity % (95% CI and *P*-value)	mean sensitivity % (95% CI and *P*-value)	AUC (95% CI and *P*-value)
A *vs* AB	55.1 (54.9–55.4 and 0.01)	52.1 (51.1–52.9 and 0.03)	55.5 (56.3–55.7 and 0.04)	0.562 (0.558–0.565 and 0.04)
A *vs* B	53.82 (53.0–53.5 and 0.03)	49.9 (49.7–50.4 and 0.03)	54.0 (53.3–54.3 and 0.01)	0.514 (0.513–0.519 and 0.03)
AB *vs* B	50.2 (49.7–50.7 and 0.04)	49.4 (48.8–50.1 and 0.03)	51.7 (51.2–52.6 and 0.04)	0.535 (0.530–0.537 and 0.03)
A *vs* O	54.9 (54.6–55.1 and 0.01)	55.9 (55.5–56.2 and 0.02)	53.6 (53.2–54.1 and 0.01)	0.557 (0.555–0.559 and 0.01)
AB *vs* O	55.2 (55.3–55.7 and 0.02)	55.8 (55.6–56.0 and 0.02)	53.1 (52.2–54.0 and 0.05)	0.568 (0.564–0.572 and 0.03)
B *vs* O	45.1 (44.9–45.3 and 0.10)	45.6 (45.4–45.8 and 0.09)	42.4 (41.6–43.1 and 0.12)	0.503 (0.500–0.507 and 0.09)

**Table 6:** Mean Values of Accuracy, Specificity, Sensitivity, and AUC of RF Models Built Comparing the Rh Blood Groups

	mean accuracy % (95% CI and *P*-value)	mean specificity % (95% CI and *P*-value)	mean sensitivity % (95% CI and *P*-value)	AUC (95% CI and *P*-value)
Rh^–^*vs* Rh^+^ blood groups	77.3 (77.1–77.5 and 0.01)	76.3 (76.2–76.5 and 0.02)	82.6 (82.1–83.6 and 0.01)	0.808 (0.808–0.810 and 0.02)

**Figure 2:**
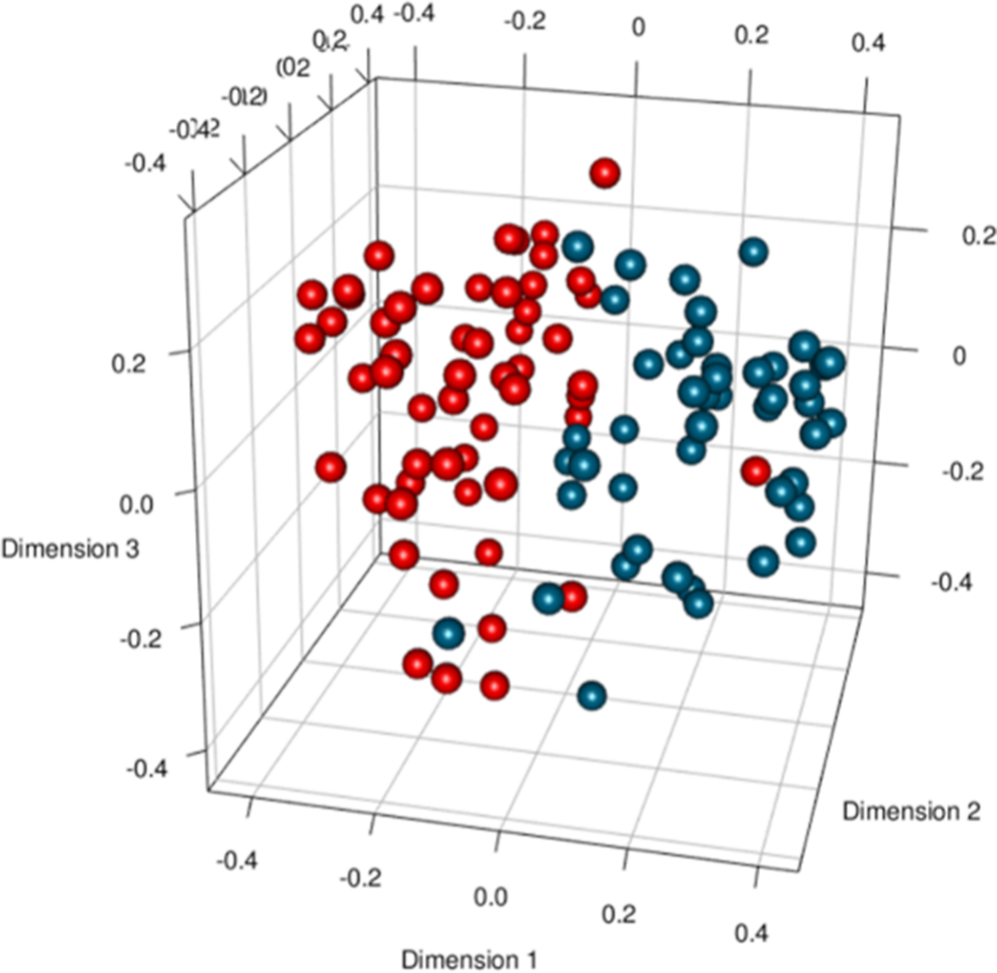
Balanced RF model score plot, taking into account the sexual dimorphism distribution. Each dot represents a single metabolic profile with colors denoting different groups of subjects. *n* = 51, randomly selected Rh^+^ blood group subjects stratified by sex (red dots); and *n* = 51, Rh^–^ blood group subjects stratified by sex (light blue dots).

**Figure 3:**
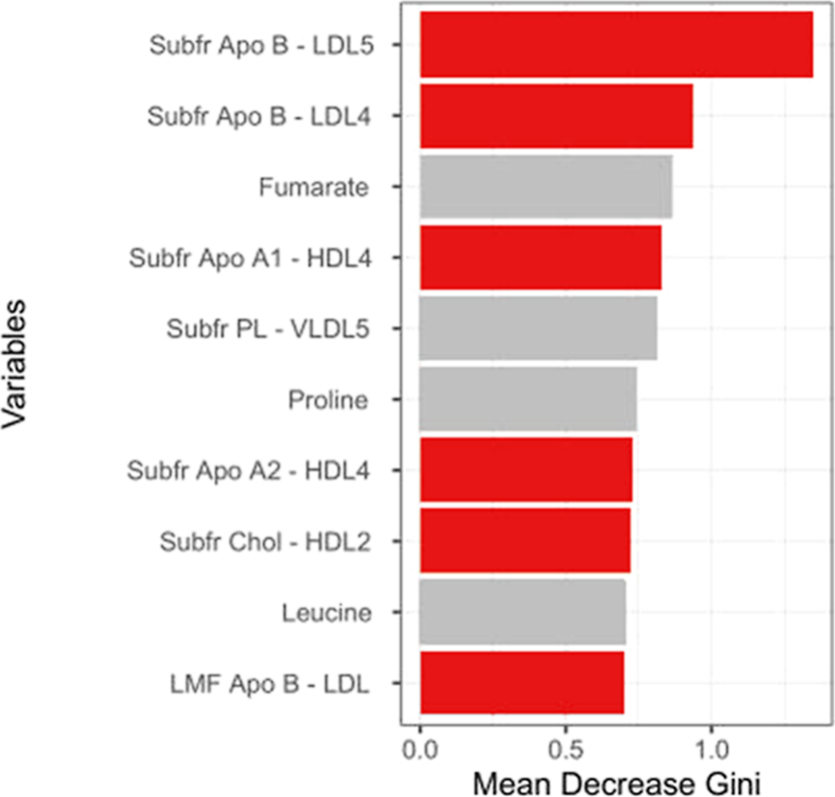
Importance of metabolites and lipoproteins in the RF model for the classification of Rh^+^ and Rh^–^ subjects calculated using the mean decrease Gini index. Gray bars correspond to no significant variables. Red bars correspond to statistically significant (false discovery rate (FDR)-adjusted *P*-value < 0.05) important variables more representative in the Rh^+^ and Rh^–^ comparison obtained with the permutation test. Only variables with a mean decrease Gini score >0.7 are shown.

In contrast, subjects with different Rh blood groups can be easily and accurately discriminated on the basis of their metabolite and lipoproteomic profiles (AUC = 0.808, *P*-value = 0.02) (see Table 6 and Figure 2). By evaluating the RF important variables, as reported in Figure 3, we observed that the subfraction LDL5 related to Apo B (Subfr. Apo B–LDL5), the subfraction LDL4 related to Apo B (Subfr. Apo B–LDL4), the subfraction HDL4 related to Apo A1 (Subfr. Apo A1–HDL4) and Apo A2 (Subfr. Apo A2–HDL4), the subfraction HDL2 related to cholesterol (Subfr. Chol–HDL2), and the lipid main fraction LDL related to Apo B (LMF Apo B–LDL) are the most relevant and significant variables in the model discriminating Rh^+^ with respect to Rh^–^ blood groups.

### 3.3 Association of ABO Blood Groups to Metabolites and Lipids

The association between the levels of circulating plasma metabolites, lipoproteins, and ABO and Rh groups was assessed by means of robust linear regression, correcting for sex and age. We observed significant association (*P*-value < 0.05) for 8 out of 114 lipoprotein fractions and subfractions with ABO groups; no significant association remains after correction for multiple testing (see Table 7). Since correction for multiple testing increases the risk of false-negative, especially in the case where (possibly) weak associations are tested on a large number of variables, we took an honest and pragmatic approach, presenting both corrected and uncorrected *P*-values and discussing the biological implications of the results for which *P*-values were significant before correction. Using robust linear modeling, we observed that the subfractions of HDL (in particular, HDL1 with a density of 1.063–1.100 kg/L and HDL2 with a density of 1.100–1.112 kg/L) and the subfraction of LDL (in particular, LDL2 with a density of 1.031–1.034 kg/L) turned out to be relevant in ABO lipidic and lipoproteomic differences. We observed non-statistically significant associations between the particle number of LDL2 (PN LDL2), the subfraction HDL1 related to Apo A1 (Subfr. Apo A1–HDL1), the subfraction HDL2 related to cholesterol (Subfr. Chol–HDL2), phospholipids (Subfr. PL–HDL2), the subfraction LDL2 related to free cholesterol (Subfr. Free Chol–LDL2), cholesterol (Subfr. Chol–LDL2), phospholipids (Subfr. PL–LDL2), and Apo B (Subfr. Apo B–LDL2) and the ABO groups. A post-hoc test on the 8 lipids and lipoproteins poorly associated with the ABO groups was also performed (see Supporting Information Table 1), highlighting that the non-statistically differences exist mainly between the A and AB, and the AB and O groups. No differences were observed between the B and O, and the A and O groups.

**Table 7:** Robust Linear Regression Models Were Performed on Serum Metabolites and Lipids of the ABO Blood Groups^a^

compound name	*P*-value	FDR *P*-value
Subfr Apo A1–HDL1	0.008	0.11
Subfr Chol–HDL2	0.01	0.11
Subfr PL–HDL2	0.02	0.11
Subfr Free Chol–LDL2	0.04	0.16
Subfr Chol–LDL2	0.04	0.16
Subfr PL–LDL2	0.04	0.16
Subfr ApoB–LDL2	0.04	0.16
PN LDL2	0.04	0.33

a Only molecular compounds with a *P*-value < 0.05 were reported; the FDR method was used for multiple testing correction. Models were adjusted for age and sex. Abbreviations used: Subfr, subfractions; Chol, cholesterol; PL, phospholipids; and PN, particle number.

It has been shown in both experimental and clinical studies that higher plasma levels of LDL and cholesterol in non-O blood groups (A, AB, B) influence the susceptibility of these groups to cardiovascular diseases, while in the O blood group, higher levels of HDL tend to play a protective role in these systemic pathologies,^46−50^ but the molecular mechanisms by which these group-specific pathologies are influenced have not yet been elucidated.

Concerning the role played by apolipoproteins (Apo), especially Apo B, there is evidence that higher numbers of RBC-bound Apo B in the O blood group compared with the non-O blood groups are associated with an atheroprotective effect and the reduction of the risk of developing vascular diseases (*i.e.*, venous thromboembolism, ischemic stroke, peripheral vascular thrombosis, and so forth).^46,51,52^

### 3.4 Association of Rh Blood Groups to Metabolites and Lipids

We observed significant associations with the Rh groups of 7 out of 114 lipoprotein fractions and subfractions and of 1 out of 27 metabolites (*P*-value < 0.05); after the correction for multiple testing, the particle number of LDL5 (PN LDL5) and the subfraction LDL5 related to Apo B (Subfr. Apo B–LDL5) were still significantly associated with Rh blood groups, as shown in Table 8. We observed associations (*P*-value < 0.05) of the lipid main fraction LDL related to triglycerides (LMF TG–LDL), Apo B (LMF Apo B–LDL), and creatine, the particle number of LDL4 (PN LDL4), and the subfraction LDL4 related to Apo B (Subfr. Apo B–LDL4) and free cholesterol (Subfr. Free Chol–LDL4) with Rh blood factors.

**Table 8:** Robust Linear Regression Models Were Performed on Serum Metabolites and Lipids of the Rh Blood Group System^a^

compound name	*P*-value	FDR *P*-value
PN LDL5	0.0008	0.01
Subfr Apo B–LDL5	0.0008	0.02
PN LDL4	0.008	0.05
Subfr Apo B–LDL4	0.008	0.05
Subfr Free Chol–LDL4	0.01	0.09
LMF TG–LDL	0.02	0.33
LMF Apo B–LDL	0.04	0.41
creatine	0.04	0.51

a Only molecular compounds with a *P*-value < 0.05 were reported; the FDR method was used for multiple testing correction. For each model built, the adjustment for age and sex was performed. Abbreviations used: Subfr, subfractions; Chol, cholesterol; LMF, lipoprotein main fractions; PN, particle number; and TG, triglycerides.

The molecular roles played by LDL4 and LDL5 in Rh^+^ and Rh^–^ group subjects have, at the best of our knowledge, never been investigated in full. One interesting observation is that the presence of the D antigen on the RBCs membrane was found to be significantly associated with lower HDL, higher triglycerides, and, in particular, higher LDL levels than the Rh^–^ group; this metabolic behavior could determine the major predisposition of Rh^+^ to develop CVDs and lipidic metabolic syndromes.^53,54^

## 4 Conclusions

The clinical significance of the ABO and the Rh blood group systems has grown beyond its use in blood transfusion and organ transplantation, and their association and correlation with various physiological and pathophysiological mechanisms have started receiving attention. In this context, to the best of our knowledge, we first present results showing the existence of specific associations between circulating levels of some plasma metabolites, lipoproteins, and the ABO/Rh blood group system in a healthy population. Using a supervised multivariate statistical approach, we were able to very weakly discriminate, using the metabolomic and lipoproteomic information, the ABO groups; in contrast, using the same approach, we were able to discriminate very well the Rh groups. We also obtained univariate associations, applying robust linear regression, between lipoproteins (especially the HDL1, HDL2, and LDL2 subfractions) and the ABO blood groups. Moreover, the LDL5 and LDL4 subfractions and creatine turned out to be significantly associated with Rh blood factors. All results highlighted how the blood groups (ABO and Rh) could be directly associated with a specific remodeling of lipoproteomic metabolism in a healthy population and can provide relevant information for further studies about the association between blood groups and disease susceptibility.

## Supporting Information Available

Post-hoc test for the regression models of ABO groups and metabolites and lipoproteins; assignment of NMR plasma spectra (PDF)

## Abbreviations

Apo: apolipoprotein
Chol: cholesterol
CF: calculated figures
CVDs: cardiovascular diseases
HDL: high-density lipoprotein
LDL: low-density lipoprotein
LMF: lipoprotein main fractions
NMR: nuclear magnetic resonance
PL: phospholipid
PN: particle number
RBCs: red blood cells
Rh: Rhesus
Subfr: subfractions
TG: triglycerides
