# Supplementary material for: Association of Plasma Metabolites and Lipoproteins with Rh and ABO Blood Systems in Healthy Subjects
Source: J Proteome Res. 2022 Oct 18;21(11):2655–63. doi: 10.1021/acs.jproteome.2c00375 (PMC9639206; doi:10.1021/acs.jproteome.2c00375)
Supplement: Supplementary file 1 — pr2c00375_si_001.pdf [file pr2c00375_si_001.pdf]

SUPPLEMENTARY MATERIAL: ASSOCIATION OF PLASMA METABOLITES AND LIPOPROTEINS WITH RH AND ABO BLOOD SYSTEMS IN HEALTHY SUBJECTS

Francesca Di Cesare[1,2], *Leonardo Tenori*[1,2,3], *Claudio Luchinat*[1,2,3], *Edoardo Saccenti*[4,*].

[1] Magnetic Resonance Center (CERM), , University of Florence, Via Luigi Sacconi 6, 50019, Sesto Fiorentino, Firenze, Italy

[2] Consorzio Interuniversitario Risonanze Magnetiche di Metallo Proteine (CIRMMP), University of Florence, Via Luigi Sacconi 6, 50019, Sesto Fiorentino, Firenze, Italy

[3] Department of Chemistry "Ugo Schiff", University of Florence, Via della Lastruccia 3, 50019, Sesto Fiorentino, Italy

[4] Laboratory of Systems and Synthetic Biology, Wageningen University & Research, Stippeneng 4, 6708 WE, Wageningen, the Netherlands.

*To whom correspondence should be addressed: Edoardo Saccenti, Laboratory of Systems and Synthetic Biology, Wageningen University & Research, Stippeneng 4, 6708 WE, Wageningen, the Netherlands. E-mail: edoardo.saccenti@wur.nl

Content:

Page 2 Supplementary Table S1

Page 3 Supplementary Figure S1

Supplementary Tables

Table S1: Robust linear regression models performed on serum metabolites and lipids of the ABO blood groups post-hoc tests. Only molecular compounds with a *P*-value < 0.05 were reported. For each comparison, estimate value, Standard Deviation (SD), *P*-value, the FDR adjusted *P*-values, and model name are also reported.

**ABO blood group comparison**	**Estimate**	**SD**	***P*-value**	**FDR *P*-value**	**Model name**
A - AB	3.18	1.08	0.02	0.08	PN LDL2
AB - O	0.82	0.81	0.02	0.08	PN LDL2
A - AB	0.15	0.35	0.02	0.08	Subfr Chol – LDL2
AB - O	-0.99	0.34	0.02	0.08	Subfr Chol – LDL2
A - AB	0.55	0.19	0.02	0.08	Subfr Free Chol – LDL2
AB - O	-0.52	0.18	0.02	0.08	Subfr Free Chol – LDL2
A - AB	0.68	0.24	0.02	0.08	Subfr PL – LDL2
AB - O	-0.65	0.23	0.03	0.10	Subfr PL – LDL2
A - AB	0.75	0.25	0.02	0.08	Subfr ApoB – LDL2
AB - O	-0.71	0.25	0.02	0.08	Subfr ApoB – LDL2
A - AB	0.33	0.09	0.003	0.05	Subfr Chol – HDL2
AB - O	-0.31	0.09	0.007	0.07	Subfr Chol – HDL2
A - AB	0.36	0.11	0.004	0.05	Subfr PL – HDL2
AB - O	-0.32	0.11	0.01	0.08	Subfr PL – HDL2
A - AB	0.91	0.25	0.002	0.04	Subfr ApoA1 – HDL1
AB - B	-0.77	0.29	0.04	0.11	Subfr ApoA1 – HDL1
AB - O	-0.91	0.25	0.001	0.04	Subfr ApoA1 – HDL1

Abbreviation used: Subfr, Subfractions; Chol, Cholesterol; LMF, Lipoprotein Main Fractions; PN, Particle Number; TG, Triglycerides

Supplementary Figures

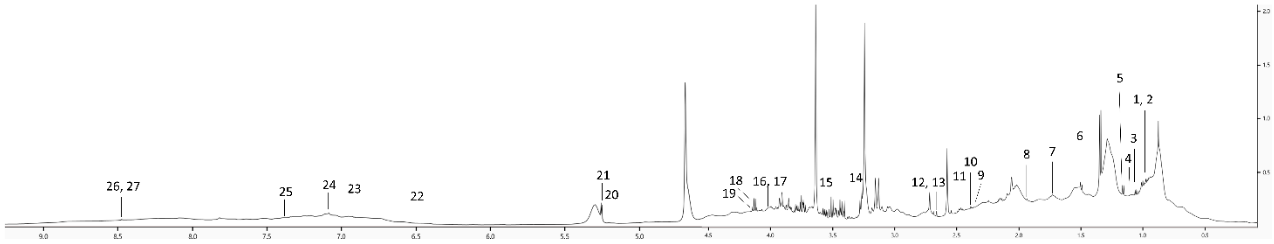

Figure S1: Assignment of a plasma ^{1}H-NOESY spectrum. 1, leucine; 2, isoleucine; 3, valine; 4, unknown 1; 5, 3-hydroxybutyrate; 6, alanine; 7, arginine+lysine; 8, acetate; 9, glutamate; 10, pyruvate; 11, glutamine; 12, methionine; 13, citrate; 14, unknown 2; 15, glycine; 16, creatine; 17, creatinine; 18, lactate; 19, proline; 20, mannose; 21, glucose; 22, fumarate; 23, tyrosine; 24, histidine; 25, phenylalanine; 26, formate; 27, adenosine nucleotide+inosine monophosphate
